# Clinical outcomes of Single-Visit oral Prophylaxis: A practice-based randomised controlled trial

**DOI:** 10.1186/1472-6831-11-35

**Published:** 2011-12-28

**Authors:** Clare L Jones, Keith M Milsom, Philip Ratcliffe, Annette Wyllie, Tatiana V Macfarlane, Martin Tickle

**Affiliations:** 1 School of Dentistry, The University of Manchester, Oxford Road, Manchester, M13 9PL, UK; 2 Department of Dental Public Health, NHS Halton & St Helens, Holloway, Runcorn WA7 4TH, UK; 3 Woodlands Dental Practice, Birkenhead, UK; 4 Martins Lane Dental Practice, Wallasey, UK; 5 Dental School, University of Aberdeen, Cornhill Road, Aberdeen, AB25 2ZR, UK

## Abstract

**Background:**

Practice-based general dental practitioners routinely provide "scale and polish" or "oral prophylaxis" to patients attending their practices. Despite its routine provision, there is no evidence to support the clinical effectiveness of single-visit scale and polish, nor the frequency at which it should be provided. A recent systematic review recommended that future trials investigating scale and polish should involve dental practice patients.

**Methods:**

A practice-based parallel randomised controlled trial with 24-month follow-up was conducted. Healthy adults (Basic Periodontal Examination [BPE] codes <3) were randomly assigned to 3 groups (6-month, 12-month, or 24-month interval between scale and polish). The primary outcome was gingival bleeding with the hypothesis that 6-monthly scale and polish would result in lower prevalence than 12-month or 24-month frequency. Follow-up measurements were recorded by examiners blinded to the allocation. 125, 122 and 122 participants were randomised to the 6-month, 12-month and 24-month groups respectively. Complete data set analyses were conducted for 307 participants: 107, 100, and 100 in the 6-month, 12-month and 24-month groups respectively. Chi-square test and ANOVA were used to compare treatment groups at follow-up. Logistic regression and ANCOVA were used to estimate the relationship between outcome and treatment group, adjusted for baseline values. Multiple imputation analyses were also carried out for participants with incomplete data sets.

**Results:**

Prevalence of gingival bleeding at follow-up was 78.5% (6-month), 78% (12-month) and 82% (24-month) (p = 0.746). There were no statistically significant differences between groups with respect to follow-up prevalence of plaque and calculus. Statistically significant differences detected in the amount (millimetres) of calculus were too small to be clinically significant. Seventeen (4.6%) participants were withdrawn from the trial to receive additional treatment.

**Conclusions:**

This trial could not identify any differences in outcomes for single-visit scale and polish provided at 6, 12 and 24 month frequencies for healthy patients (with no significant periodontal disease). However, this is the first trial of scale and polish which has been conducted in a general practice setting and the results are not conclusive. Larger trials with more comprehensive measurement and long-term follow up need to be undertaken to provide a firm evidence base for this intervention. This trial informs the design of future practice-based trials on this subject.

## Background

General dental practitioners (GDPs) routinely recommend and provide "scale and polish" or "oral prophylaxis" to patients attending their practices [1,2]. This single-visit treatment consists of supra- and sub-gingival scaling to remove hard deposits, and polishing the teeth with a powered cup, or brush, and paste.

Different definitions of the term 'routine scale and polish' exist and its role in the management of periodontal disease is not specifically defined [3]. Scale and polish is intended to complement patients' self-care plaque-control methods and historically has become inextricably linked to the routine (six-monthly) dental check-up, even if a patient has no, or low, risk of developing periodontal disease [2-5]. Oral hygiene instruction may be provided in conjunction with the scale and polish to encourage positive oral health behaviour change and improved self-care; furthermore, it has been suggested that there is little value to the professional intervention if hygiene advice is not given [6].

Provision of single-visit scale and polish incurs costs for privately-paying patients and for tax-payers in publicly-funded healthcare systems. It is unclear what proportion of this treatment is clinically necessary [7], yet in 2009/10, 12 million (44.1% of total) courses of treatment carried out on adult National Health Service (NHS) patients in England included a scale and polish [1].

Despite its routine provision, there is a debate regarding the clinical effectiveness of single-visit scale and polish, and the frequency at which it should be provided. A systematic literature review [3] was unable to reach firm conclusions about the beneficial effects on periodontal health and recommended (practice-based) randomised controlled trials (RCTs) to investigate the effectiveness of the intervention.

This paper reports the findings of a preliminary RCT which aimed to compare gingival health outcomes of single-visit scale and polish, performed at 6-, 12- or 24-month intervals, in healthy adults, with no significant periodontal disease who were regular attenders at 'family' dental practices. The objectives were to compare presence of gingival bleeding, dental plaque, and amount of calculus between groups receiving single-visit scale and polish at these intervals.

## Methods

The trial protocol responded to recommendations [3] and was reviewed and approved by Cheshire Local Research Ethics Committee (reference: Q/1506/100.) The trial was registered with UKCRN: (ID5101); and ISRCTN (ISRCTN56889016). The Oral Health Unit at The University of Manchester funded the trial. Research support costs were met by Cheshire and Merseyside Comprehensive Research Network (funded by the National Institute for Health Research.)

The study was a randomised, 3-arm, parallel clinical trial with an allocation ratio of 1. The follow-up period was 24 months; the maximum period advised between dental check-ups by national guidelines [8].

### Participants

Participants were recruited from three multi-surgery family dental practices in Northwest England that had sufficient estate space and large patient populations, enabling them to host the trial. Regularly-attending patients aged 18-60 years who were scheduled for a dental check-up were sent an appointment for a dedicated trial recruitment session with written information about the trial i.e. specific days/sessions were set aside for trial recruitment rather than patients being recruited on an ad hoc basis when they attended for their routine dental appointment. On attendance the trial was discussed with patients and informed, written consent was obtained by a member of the research team. Participants were free to withdraw from the trial at any time, without explanation.

**Figure 1:**
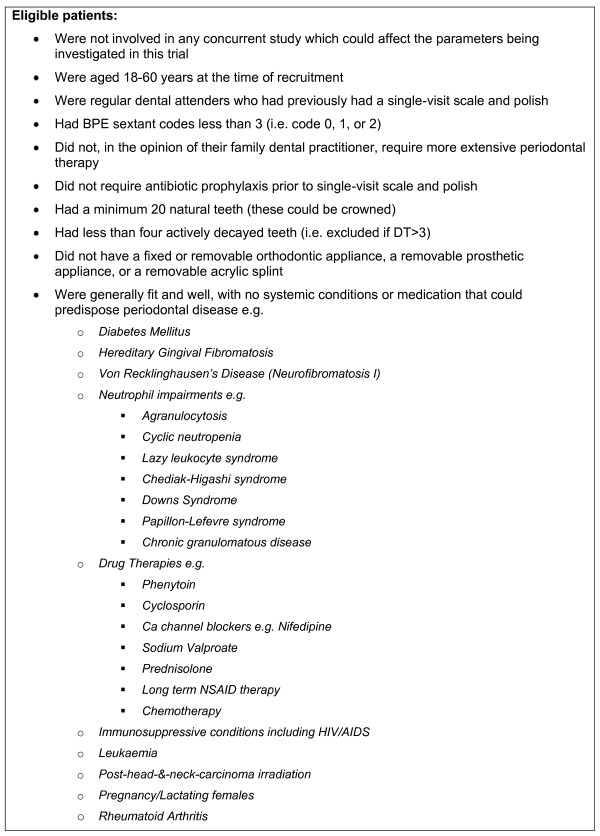
**Participant Trial Inclusion Criteria**.

Eligibility checks were carried out by the patients' own GDPs using standardised pro forma and by two independent trial examiners. The latter were registered dentists who worked in the salaried dental services and who had no connection to the trial practices. Full details of the inclusion criteria are presented in Figure 1. The principal exclusion criteria were BPE code 3/4/* (*see *Additional File 1) in one or more sextants [9] and evidence of systemic periodontal risk factors [10]. It was thought that confining the inclusion criteria to non-smokers may have compromised recruitment given that it is not an uncommon habit. Smokers were therefore not excluded as long as they fulfilled the eligibility criteria.

### Sample Size & Randomisation

Data available to inform the sample size calculation was limited. A pragmatic approach was taken and a suite of power calculations was carried out with advice from a specialist in periodontology (*see *Additional File 2). Assuming 20% loss to follow-up 369 participants were required to achieve a sample size of 96 per group at follow-up. This was sufficient to detect a clinically significant difference in proportions of bleeding (power 90%; α = 0.01) assuming a 30% bleeding rate in the 6-month group, 45% in the 12-month group and 60% in the 24-month group.

Baseline assessment was undertaken by the two independent trial examiners prior to randomization of the 369 recruited participants to ensure allocation concealment. This examination also enabled participants to be stratified according to presence/absence of supra-gingival calculus prior to randomization. Treatment allocation was by minimization [11] and carried out by the trial manager using MINIM, an MS-DOS program [12].

Participants' group allocation was not revealed until they returned for their first 6-month recall; they were informed of this by the hygienist providing the trial intervention. Participants' family dentists were blind to intervention allocation in as much as this was not revealed by the research team; participants were asked not to disclose their allocation group to their dentist or to the outcome examiner. The same two examiners carried out all of the follow-up examinations blind to the allocation.

### Interventions

Single-visit scale and polish treatments were carried out by 9 hygienists and therapists employed by the dental practices. All had appropriate professional qualifications, and were registered with the UK professional regulatory body (General Dental Council.) A standard definition of single-visit scale and polish [3] was adopted: hygienists and therapists were instructed to carry out supra- and subgingival scaling, or polishing, or both, of the crown and root surfaces of teeth to achieve an end-point of no deposits and/or staining. There was no adjunctive root planing or chemotherapeutic therapy and local anaesthetic was not used. An ultrasonic scaler and an air motor-driven rotary rubber cup with polishing paste were used unless participants were unable to tolerate ultrasonic instrumentation; in such cases, hand-scaling was performed.

**Figure 2:**
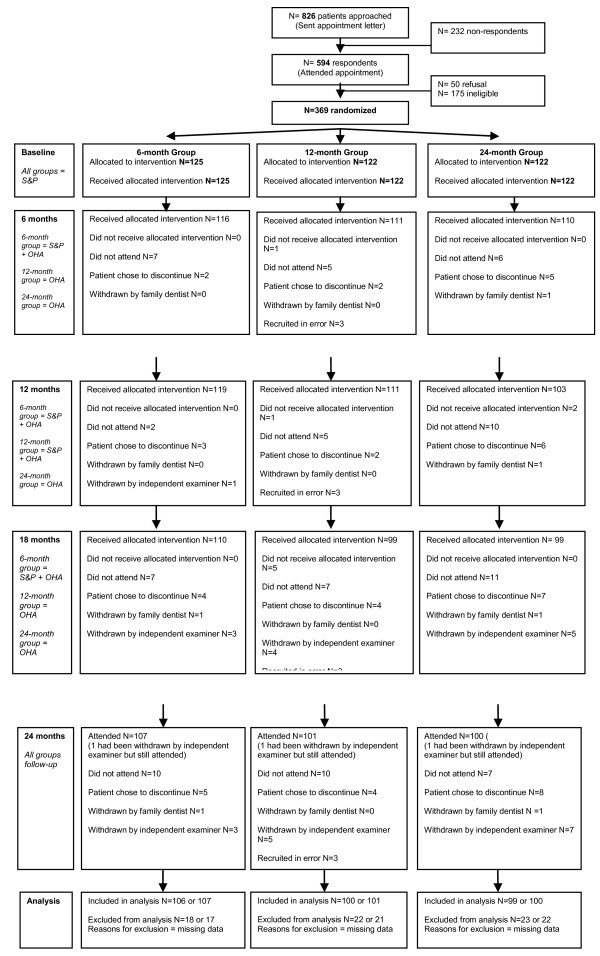
**RCT CONSORT flow diagram (Recruitment, Baseline, 6 months)**.

**Figure 3:**
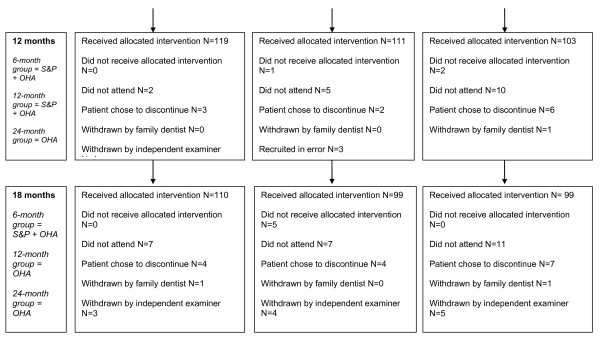
**RCT CONSORT flow diagram (12 months, 18 months)**.

**Figure 4:**
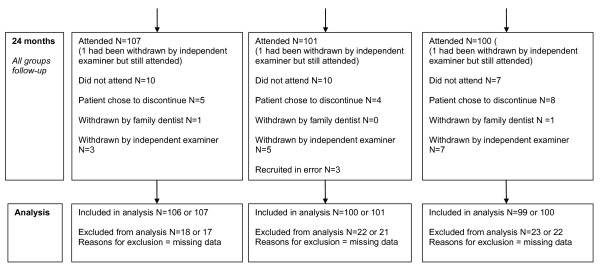
**RCT CONSORT flow diagram (24-month follow-up, Analysis)**.

All participants received a baseline single-visit scale and polish after baseline assessment. Throughout the 24-month follow-up period, all participants were recalled every 6-months for routine examination with their family dentist which included monitoring of their periodontal condition using BPE [9,13]. If practitioners had concerns about a participant's periodontal condition, they were referred to one of the two independent trial examiners. If the independent trial examiner detected a BPE code 3, this led to the participant being withdrawn from the trial to receive appropriate treatment. At baseline and at each subsequent 6-monthly appointment all participants received standardised oral hygiene advice from a hygienist [14]; (*See *Additional File 3.) The same hygienist delivered the intervention: the 6-month group received a single-visit scale and polish at 6, 12, and 18-months; the 12-month group received single-visit scale and polish at 12-months. The 24-month group received no scale and polish interventions after baseline for the duration of the trial. Participants were allocated 15-20 minute appointments for oral hygiene advice plus intervention; however, additional time was permitted, as required, to complete the intervention within a single-visit. A diagrammatical representation of the trial is provided in Figures 2, 3, 4.

The hygienist trial record sheets were reviewed at the end of each session. If a participant was scheduled to have a scale and polish at a recall session had erroneously not received this, every effort was made to contact them to arrange another appointment so that this could be delivered.

### Outcomes

The primary outcome measure was presence of gingival bleeding (dichotomous); secondary outcomes were presence of plaque (dichotomous), presence (dichotomous) and amount (millimetres) of calculus.

Outcome measurements were taken at baseline and 24-month follow-up by two independent examiners. The baseline examination was undertaken before the delivery of the baseline scale and polish. The outcome measurements were taken before the participants saw their own family dentist for a check up and provision of any scaling and polishing deemed necessary i.e. measurements were taken approximately 6 months after the 18-month intervention. Both examiners were experienced in examining for national epidemiological studies [15]. For the purposes of this trial, they undertook training (*See *Additional File 4) in the examination procedure prior to baseline and follow-up examinations. The following information was recorded for each participant:

• Bleeding from the gingival margin of six (Ramfjord) index teeth [16,17]. Bleeding was detected by running a blunt-ended (PCP-10) probe gently around the gingival margin of the tooth at a 60° angle, in contact with the sulculur epithelium. After approximately 30 seconds, any bleeding elicited was recorded according to a dichotomous scale for each tooth: present/not present.

• Visual presence of any plaque on the same index teeth according to a dichotomous scale: plaque present/not present.

• Measurement of calculus in millimetres: One measurement, confined to the lingual surfaces of the mandibular incisor and canine teeth. A PCP-10 probe was used to measure along the vertical axis of the tooth with the most calculus.

### Analysis

Statistical analysis was conducted by the trial statistician (TM) blind to the allocation, i.e. the treatment groups were coded without disclosing the labelling. PASW Statistics 18 [18] and STATA [19] were used for data analysis.

Demographic baseline characteristics were described. Hypothesis testing of baseline imbalance is not recommended practice and, therefore, was not performed [20-22]

Complete case analysis was carried out initially. Intention to treat (ITT) analysis [23] was not possible as 100% follow-up was not achieved. To minimise bias caused by missing data, multiple imputation was employed for participants with incomplete datasets [24].

A Chi-square test was used to compare dichotomous data in treatment groups at follow-up: prevalence (presence) of any bleeding, any plaque and any calculus by participant (rather than by tooth). The proportion of teeth with bleeding at follow-up was calculated as the total number of teeth with any bleeding divided by the total number of teeth examined for bleeding. The proportion of teeth with plaque at follow-up was calculated in a similar manner. An analysis of variance (ANOVA) was used to compare treatment groups at follow-up for:

• proportion of teeth with bleeding;

• proportion of teeth with plaque; and

• mean amount (millimetres) of calculus.

Logistic regression and ANCOVA were used to estimate the relationship between outcome and treatment group, adjusted for the baseline values.

Multiple imputation (n = 100 imputations) was performed using *mi logit *and *mi mvn *procedures in STATA [19]. Variables used in imputation were: baseline values, gender, baseline age, deprivation score and randomization group. Deprivation was calculated using the Index of Multiple Deprivation; a small area measure derived from participants' residential postcodes [25]. The 6-month group represented traditional frequency of scale and polish and was used as the reference group to which other groups were compared.

## Results

The CONSORT flow diagram [20] is presented in Figures 2, 3, 4. There were 40 dedicated recruitment sessions 02/2006 to 09/2007. Of the 826 patients approached, 44.7% (N = 369) consented, and were randomly allocated to a trial group. Of the 369 participants commencing the trial, 3 were found to have been recruited in error and did not fulfil the inclusion criteria. Seventeen participants chose to discontinue the trial (5 from 6-month group, 4 from 12-month group, and 8 from 24-month group.) Two participants were withdrawn by their family dentist and a further fifteen were withdrawn from the trial by the independent trial examiners due to concerns that they had a BPE code of 3 (Total 17: 6 from 6-month group; 4 from 12-month group; 9 from 24-month group.). Follow-up data were collected for 83.5% of the original participants; 76.2% attended all 5 trial appointments. Baseline demographic and clinical characteristics of trial participants are presented in Table 1.

**Table 1:** Baseline demographic and clinical characteristics of trial participants

Characteristic	6-month Group	12-month Group	24-month Group
**Baseline No. of Participants**	125	122	122

**Age (years)**			
Mean (SD)	37.1 (10.4)	39.6 (10.8)	36.4 (10.6)

**Gender**			
N (%) Male	57 (45.6)	43 (35.2)	34 (27.9)

**IMD Quintile^a^**			
N (%)			
1 Most Deprived	40 (32.0)	40 (32.8)	34 (27.9)
2	29 (23.2)	29 (23.8)	30 (24.6)
3	18 (14.4)	18 (14.8)	24 (19.7)
4	24 (19.2)	21 (17.2)	21 (17.2)
5 Least Deprived	14 (11.2)	14 (11.5)	13 (10.7)

**Smoking history^b^**			
N (%)			
Never	83 (66.4)	70 (57.4)	71 (58.2)
Past	21 (16.8)	31 (25.4)	29 (23.8)
Current	12 (9.6)	15 (12.3)	15 (12.3)
Missing	8	6	7

**No. of Teeth present**			
Mean (SD)	27.8 (2.4)	27.8 (2.1)	27.6 (2.3)
Missing	0	1	0

**Decayed Teeth**			
N (%) any	9 (37.5)	8 (33.3)	7 (29.2)
Missing	0	1	0

**Filled Teeth**			
Mean (SD)	7.7 (4.5)	7.7 (4.7)	6.8 (4.3)
Missing	0	1	0

**Baseline clinical data for participants attending both baseline and follow up examinations**			

**Bleeding prevalence N = 307**			
N(%) with any bleeding	54 (50.5)	63 (63.0)	65 (65.0)

**Proportion of index teeth with bleeding N = 307**			
Mean (SD)	17.4 (21.6)	21.2 (21.0)	21.5 (21.5)

**Plaque prevalence N = 307**			
N (%) with any plaque	81 (75.7)	79 (79.0)	77 (77.0)

**Calculus prevalence N = 305**			
N (%)with any calculus	64 (60.4)	53 (53.5)	52 (52.0)

**Amount Calculus (mm) N = 305**			
Mean (SD)	0.79 (0.77)	0.80 (0.94)	0.72 (0.83)

^a^IMD derived from participants' postcodes. Quintiles based on national standards.

^b^Self reported smoking status based upon response to the following questions: Do you currently smoke? (yes/no); Have you ever smoked? (yes/no) For participants recruited 02/2006 - 09/2006 these data were reported retrospectively, at the 12-month recall. For patients recruited 2007, smoking data were reported at baseline.

Prevalence of gingival bleeding increased in all groups between baseline and follow-up (Table 2). There were no significant differences between groups at follow-up (P = 0.746). Odds ratios, adjusted for baseline bleeding, showed no significant association between frequency of scale and polish and bleeding prevalence.

**Table 2:** Effect of Scale and Polish on Gingival Bleeding

	6-month Group	12-month Group	24-month Group	Statistical Test
**Prevalence of gingival bleeding**				

**Data available for both baseline and follow up (N = 307)**	107	100	100	

N (%) with any bleeding at follow-up	84 (78.5)	78 (78.0)	82 (82.0)	Χ^2 ^test P = 0.746

**Complete data analysis (N = 307)**				

Odds Ratio (from Logistic regression) (95% CI) for follow up adjusted for baseline bleeding	1.00	0.92 (0.47, 1.79)	1.17 (0.59, 2.35)	

**Multiple imputation analysis^c ^(N = 368)**				

Odds Ratio (from Logistic regression) (95% CI) for follow up adjusted for baseline bleeding	1.00	0.92 (0.45, 1.89)	1.19 (0.58, 2.47)	

**Proportion of index teeth examined with gingival bleeding^d^**				

**Complete data analysis (N = 307)**				

**Follow-up % of index teeth with bleeding** Mean (SD)	37.9 (30.3)	38.8 (30.7)	39.8 (30.2)	ANOVA P = 0.896

**Follow-up % of index teeth with bleeding adjusted for baseline bleeding**				ANCOVA P = 0.979

**Multiple imputation analysis^c ^(N = 368)**				

**Follow-up % of index teeth with bleeding adjusted for baseline bleeding** **(from linear regression**)				P = 0.932

^c^Follow-up data were imputed using baseline values, gender, age at baseline, deprivation score and randomization group.

^d^The percent of index teeth with bleeding was calculated for each participant as the total number of teeth with bleeding over the total number of index teeth examined.

There were no significant differences between groups at follow-up with respect to prevalence of plaque (P = 0.183) and calculus (P = 0.615) (Table 3).

**Table 3:** Effect of Scale and Polish on Plaque and Calculus

	6-month Group	12-month Group	24-month Group	Statistical Test
**Prevalence of plaque**				

**Data available for both baseline and follow up (n = 307)**				

N (%) with any plaque at follow-up	79 (73.8)	76 (76.0)	84 (84.0)	Χ^2 ^test P = 0.183

**Complete data analysis (N = 307)**				

Odds Ratio (from Logistic regression) (95% CI) for follow up adjusted for baseline plaque	1.00	1.08 (0.57, 2.07)	1.89 (0.93, 3.81)	

Multiple imputation analysis* (N = 368)				

Odds Ratio (from Logistic regression) (95% CI) for follow up adjusted for baseline plaque	1.00	1.04 (0.54, 1.98)	1.90 (0.93, 3.86)	

**Proportion of index teeth with plaque. Data available for both baseline and follow-up (N = 307)**				

**Follow-up % of index teeth with plaque** Mean (SD)	39.4 (34.2)	43.5 (34.7)	43.7 (32.4)	ANOVA P = 0.587

**Follow-up % of index teeth with plaque adjusted for baseline plaque**				ANCOVA P = 0.597

Multiple imputation analysis* (N = 368)				

**Follow-up % of index teeth with plaque adjusted for baseline values (from linear regression**)				P = 0.653

**Prevalence of lingual calculus**				

**Data available for both baseline and follow up (N = 305)**				

N (%) with any calculus at follow-up	59 (55.7)	54 (54.5)	61 (61.0)	Χ^2 ^test P = 0.615

**Complete data analysis (N = 305)**				

Odds Ratio (from Logistic regression) (95% CI) for follow up adjusted for baseline calculus	1.00	1.10 (0.60, 2.04)	1.58 (0.85, 2.95)	

Multiple imputation analysis* (N = 367)				

Odds Ratio (from Logistic regression) (95% CI) for follow up adjusted for baseline calculus	1.00	1.12 (0.60, 2.09)	1.64 (0.86, 3.13)	

**Amount (mm) of lingual calculus**				

**Data available for both baseline and follow up (N = 305)**				

**Follow-up Calculus** Mean (SD)	0.71 (1.00)	0.89 (0.99)	0.95 (0.97)	ANOVA P = 0.022 *Mean difference (95% CI)* *6-24: -0.32 (-0.61, -0.02)* *6-12: -0.26 (-0.55, 0.04)* *24-12: 0.06 (-0.24, 0.36)*

**Follow up Calculus adjusted for baseline calculus**				ANCOVA P = 0.001

**Multiple imputation analysis (N = 367)**				

**Follow-up Calculus adjusted for Baseline values (from linear regression**)				P < 0.001

The mean amount (height) of calculus present on the lower anterior teeth at follow-up was: 6-month group 0.71 mm (SD 1.00); 12-month group 0.89 mm (SD 0.99); 24-month group 0.95 mm (SD 0.97). Adjustment of follow-up calculus for baseline measures showed this difference to be statistically significant (ANCOVA p = 0.001).

The results of the multiple imputation analysis were similar to the complete dataset analysis: no additional statistically significant differences were identified.

Ancillary analyses examined the proportion of index teeth with bleeding or plaque. All groups demonstrated increased prevalence in bleeding from baseline to follow-up. Follow up proportions of teeth with bleeding (Table 2), were 37.9% (SD 30.3) in 6-month group; 38.8% (SD 30.7) in 12-month group; 39.8% (SD 30.2) in 24-month group. These proportions were not significantly different between groups (ANCOVA p = 0.979)

Follow-up proportions of teeth with plaque were 39.4% (SD 34.1), 43.0% (SD 34.9), and 43.7% (SD 32.4) in the 6-month, 12-month and 24-month groups respectively. Differences between groups were not significant (ANCOVA P = 0.597.)

## Discussion

This is the first randomised control trial in the literature investigating the effectiveness of scale and polish when it is delivered in a general practice setting. It should therefore be seen as the first stepping stone to improve the evidence-base for this commonly provided intervention rather than providing definitive evidence to inform policy on resource allocation or as a basis for clinical guidelines. This was a pragmatic trial, involving healthy adult participants with no significant periodontal disease, who had a history of regularly visiting their family dental practitioner. It was not an explanatory trial and did not investigate an intervention performed on patients with periodontal disease or under specialist care. Over a 24-month follow-up period, the researchers could not detect a statistically or clinically significant difference in gingival health measures between groups. The difference in the amount of supragingival calculus between groups was statistically significant yet too small to be clinically significant, given that it is unlikely that it could be detected clinically by family dentists using the instruments commonly used in general dental practice. Prevalence of plaque at baseline (and follow-up) was comparable to findings of national epidemiological surveys [26]. This suggests that the trial has reasonable external validity and that even in a motivated population presence of plaque is the 'norm'. The overall increase in the primary outcome measure trial across the whole trial population gives cause for concern. One interpretation of this finding could be that it is due to a real deterioration in gingival health across all three groups. Furthermore this decline could be attributed to sub-optimal delivery of the scale and polish. This could well be the case, but equally the dental care professionals (hygienists and therapists) could have striven to provide a more thorough scale and polish than usual. Knowing that their work would be independently assessed, it could be argued that this latter scenario is more likely than providing sub-optimal care. In pragmatic trials there will be greater variation in the delivery of the intervention under test than is the case for explanatory trials and therefore the intervention is less likely to be effective. In addition, for interventions for which the practitioner cannot be blinded to the allocation, as is the case in many trials of surgical interventions, there will always be the risk of a Hawthorne effect [27] resulting in the intervention under test being not truly representative of the intervention as it is provided in 'real life'. In this trial participants were monitored by their 'family dentist' every 6 months and dental practitioners could refer participants giving cause for concern to an independent examiner who was blind to the allocation. A small number of patients were referred and a smaller number removed from the trial because of clinical concerns. This and the relative stability of plaque scores between baseline and follow up suggest that inter examiner variation in probing force used to assess gingival bleeding is a more likely to account for the increase in bleeding on probing rather than an overall deterioration in gingival health of the trial population. This interpretation is supported by analyses which demonstrate that examiner 1 assessed more patients at baseline and examiner 2 assessed more patients at follow up (Table 4). The overall increase in bleeding could be explained by examiner 2 using a consistently greater probing force than examiner 1. However there were no significant differences in the distribution of participants examined by the two examiners at baseline and follow up across the three arms of the study and therefore inter-examiner variation would have a minimal influence on the between-group findings.

**Table 4:** Distribution of examiners at baseline and follow-up by treatment group

Characteristic	6-month Group N (%)	12-month Group N (%)	24-month Group N (%)	Statistical Analysis
Baseline and Follow-up both examiner 1	28 (26.2)	25 (25.0)	27 (27.0)	Χ^2 ^test P = 0.995
	
Baseline and Follow-up both examiner 2	10 (9.3)	9 (9.0)	10 (10.0)	
	
Baseline examiner 1; Follow-up examiner 2	69 (64.5)	66 (66.0)	63 (63.0)	

Significant barriers to executing research in general practice have been reported [28,29], and were experienced in the operational management of this trial. Lack of empirical data on the expected effect size to inform a power calculation meant that the sample size was based on estimates according to clinical expectations and an ability to identify an effect size which would influence clinicians to consider changing their clinical practise, Recruitment of participants was logistically difficult and caused considerable disruption to day-to-day running of the dental practices, potentially effecting practice income; for this reason clinical measurement was limited and recruitment ceased once the trial had achieved its recruitment target. A larger sample size would have resulted in tighter confidence intervals and the authors acknowledge that a longer follow-up period would have been desirable; a 5-year period [3] was initially proposed but a compromise (2-year follow-up) was reached in response to concern expressed by family dentists about withholding treatment for such a long period. On a positive note, participant loss to follow-up was low (less than 20%) and this preliminary trial demonstrates that dental practices and dental patients can be successfully recruited to practice based trials; with significantly greater resources larger sample sizes and longer follow up periods would be possible.

Interpretation of trials with non-significant findings is difficult, particularly for preliminary trials because bias tending towards a non-significant result can stem from different sources. The potential for allocation bias was considered to be small as the trial manager had no knowledge of the patients other than the basic details required for randomization. Baseline imbalances therefore occurred by chance rather than allocation bias; the magnitude of chance imbalance was not considered to be clinically significant. The 24-month group had a lower proportion of males than other groups, but gender is not a significant risk for periodontal disease progression [30]; and similar proportions of males and females were lost to follow up. Whilst smoking is a risk factor for periodontal disease, smokers were included if they fulfilled the eligibility criteria. The randomisation process ensured that smokers were evenly distributed between groups. Subgroup analysis of the smokers was not undertaken due to the small numbers and because inadequately powered post hoc analyses can produce misleading results [31]. Future trials on this topic may wish to confine the trial population to smokers or power the trial to enable well designed sub-group analyses.

### Pragmatic trials vs. explanatory trials

This was a pragmatic trial i.e. one which primarily seeks to determine the effects of an intervention under the usual conditions in which it will be applied; in contrast, explanatory trials are primarily designed to determine the effects of an intervention under ideal circumstances [32]. The continuum between explanatory trials and pragmatic trials is very helpfully examined by Thorpe et al. [33] who identify variation in practitioner adherence to applying the intervention (discussed above), and patient compliance with the intervention as factors which reduce the effect size of interventions tested in pragmatic trial. Pragmatic trials also tend to have greater flexibility in eligibility criteria, (usually far less stringent than explanatory trials), less intensive follow up and greater flexibility in applying the experimental and comparison interventions. As a consequence a smaller effect size or even a non-significant result is a more likely finding in pragmatic trials than explanatory trials because of the 'noise' around the process of delivery of the intervention. This means that future pragmatic trials in this field will require much larger numbers to detect the 'signal' of the intervention delivered in a real world practice setting.

There were also issues around the outcome measures used in this trial. Guidance suggests that periodontitis is best assessed long-term by measuring attachment levels, but cautions that any change less than 2 mm could be due to measurement error [34]. It must be remembered that the trial population consisted of patients without significant periodontal disease (BPE < 3) who attend family dental practices on a regular basis; the measures used were chosen as they are simple clinical indicators which would prompt action by a GDP and change the care plan for patients [4]. The same two experienced examiners were used at both baseline and follow up, and both received the same extensive training by a specialist periodontologist prior to baseline and follow-up examinations. However, there was the possibility of inter-examiner variation as it was not possible to calibrate examiners given the invasive nature of the examination. The trial reflects the tensions felt, particularly in pragmatic trials, between using very precise, interval scale measures which increase the power of trials, and using dichotomised outcomes which decrease power but are intuitively more meaningful to GDPs and patients. So the measures used in this preliminary trial were limited and in future trials a more comprehensive approach to outcome measurement should be used. Trialists working in the dental field should consider how to reach a consensus on trial outcome measures perhaps under the COMET (Core Outcome Measures in Effectiveness Trials) Initiative [35] which seeks to obtain consistency in the choice of outcome measures for trials. However, in this field there would still be concerns about inter examiner reliability particularly in very large pragmatic trials with multiple examiners.

Whilst the trial participants were asked not to reveal their group allocation, evidently, there was potential for them to inform the independent examiners. Furthermore, it is possible that participants' clinical appearance may have been indicative of trial group. This is acknowledged as a limitation of the trial; and whilst one could argue that bias due to inadequate allocation concealment in this trial would work in the direction of finding a significant difference between groups, this risk should be minimised in the design of trials and through careful management. Questioning examiners to determine whether they could identify group allocation may have enabled researchers to identify bias with respect to reporting outcome measurements [36].

One source of bias which could tend to a non-significant finding is the possibility that group allocation could have selectively affected loss to follow-up. In this trial there was greater loss to follow-up in the 24-month group than the 12- and 6-month groups, although the difference in numbers was small (The proportions of original participants who attended 24-month follow-up were: 85.6% 6-month group; 82.8% 12-month group; 82.0% 24-month group.) Selective loss to follow-up is always a potential problem for pragmatic, non-blinded trials as patients are likely to have a preference for the 'usual' treatment (a commonly used control in pragmatic trials) or for a new treatment under test. The potential for which way the bias is directed (towards a significant or a non-significant finding) is, we suspect, specific to each trial. A parallel qualitative component of the trial design to assess participant's views of the relative merits of experimental and comparison interventions may be helpful in assessing the risk of bias of selective loss to follow up.

There is no single indicator which accurately predicts healthy patients' risk of periodontal disease. Chronic gingivitis [37], smoking, poor oral hygiene, and being over 65 years of age [38] are all predictors. It could be argued that routine 6-monthly scale and polish represents a practice-based population approach to preventing periodontal disease. However, if there really is no difference in gingival health outcomes when this intervention is provided less frequently, routine 6-monthly treatment for low risk patients is difficult to support. It should be remembered that routine provision of scale and polish for healthy patients has opportunity costs for state-funded healthcare systems i.e. time which could be spent performing more clinically effective treatments. This may not be sustainable in the current economic climate. There are also issues with respect to informed decision making for privately-paying patients with no significant periodontal disease. However this trial is preliminary and therefore cannot provide firm evidence to inform policy or clinical practise.

The importance of oral hygiene instruction in conjunction with scale and polish delivery was not investigated in this RCT. There is some evidence from non-practice based trials which suggests that the professional intervention to remove plaque in conjunction with oral hygiene instruction is as effective as oral hygiene instruction on its own; and that both of these are more beneficial than no treatment at all [6]. This however is a different research question than the one asked in this trial, which was concerned with frequency of scale and polish delivery. A larger sample size is required to investigate groups receiving scale and polish at different frequencies with or without oral hygiene advice, and the constraints of this trial meant that this was not feasible. There is therefore a further requirement for future practice-based research to investigate the contributions of scale and polish and oral hygiene instruction to periodontal health alone and in combination. Two of the authors (CJ, MT) are involved with just such a practice-based trial which will seek to unpick this relationship [39].

## Conclusions

Single-visit scale and polish is a treatment historically embedded in general dental practice rather than being a defined treatment for periodontal disease. The question of whether or not routinely providing a 6-monthly single-visit scale and polish is an effective use of professional resources for healthy adults with no significant periodontal disease cannot be answered by the first practice-based trial in the literature. This preliminary trial does not provide sufficient evidence to support or refute the benefit of 6-month scale and polish over 12- or 24-month treatment provision; it does however raise important questions and will inform the design and conduct of further pragmatic practice-based trials which seek to investigate this subject.

## Abbreviations

ANOVA: Analysis of Variance; ANCOVA: Analysis of Covariance; BPE: Basic Periodontal Examination; CI: Confidence Interval; DCP: Dental care professional; GDP: General dental practitioner; IMD: Index of Multiple Deprivation; NHS: National Health Service; NIHR: National Institute for Health Research; OHA: Oral Hygiene Advice; OR: Odds Ratio; RCT: Randomised controlled trial; S&P: Single-visit scale and polish; SD: Standard deviation.

## Competing interests

The authors declare that they have no competing interests.

## Authors' contributions

CJ was responsible for the methodological design of the trial, drafted the study protocol, managed the delivery of the trial, collected and managed the trial data, drafted the manuscript, and carried out subsequent revisions. PR contributed to the development of the protocol and the operational management of the study, including participant recruitment. AW contributed to the development of the protocol and the operational management of the study, including participant recruitment. TM was responsible for the methodological design of the trial, drafted the study protocol, designed and conducted the statistical analyses, and commented on drafts of the manuscript. KM was responsible for the initial research question, the methodological design of the trial, drafted the study protocol, and commented on drafts of the manuscript. MT was responsible for the initial research question, the methodological design of the trial, drafted the study protocol, and drafted the manuscript. All authors provided comments on the drafts and have read and approved the final version. The views and opinions expressed are those of the authors and do not necessarily reflect those of the NIHR, NHS or the Department of Health.

## Pre-publication history

The pre-publication history for this paper can be accessed here:

http://www.biomedcentral.com/1472-6831/11/35/prepub

## Supplementary Material

Additional File 1 **Basic Periodontal Examination details**. Click here for file

Additional File 2 **Sample size calculation information**. Click here for file

Additional File 3 **Delivery of standardised oral hygiene advice**. Click here for file

Additional File 4 **Details of training for independent trial examiners in outcome measurements**. Click here for file
