# Supplementary material for: Clinical outcomes of Single-Visit oral Prophylaxis: A practice-based randomised controlled trial
Source: BMC Oral Health. 2011 Dec 28;11:35. doi: 10.1186/1472-6831-11-35 (PMC3280181; doi:10.1186/1472-6831-11-35)
Supplement: Additional File 1 — Basic Periodontal Examination details. [file 1472-6831-11-35-S1.DOC]

Basic Periodontal Examination Codes

| Code | Description | Treatment need |
| --- | --- | --- |
| 0 | A sextant in which there are no pockets in excess of 3.5mm deep, there are no overhanging restoration margins or calculus, and no bleeding after gentle probing. | No treatment required  Screen again in 12 months |
| 1 | A sextant in which there are no pockets in excess of 3.5mm deep, there are no overhanging restoration margins or calculus, but bleeding occurs after gentle probing. | Oral hygiene instruction and prophylaxis  Screen again in 12 months |
| 2 | A sextant in which there are no pockets in excess of 3.5mm deep (i.e. the coloured band of the probe remains visible) but plaque retentive factors are seen or recognised underneath the gingival margin (e.g. overhanging restoration margins or subgingival calculus) | Oral hygiene instruction, and prophylaxis. Supra and sub-gingival scaling Remove overhanging restoration etc  Screen again in 12 months |
| 3 | A sextant in which the coloured band of the probe (3.5mm to 5.5mm) remains partially visible when it is inserted into the deepest pocket. | Plaque distribution gingival inflammation and probing depths to be recorded in sextant(s) at completion of treatment.  Treatment of sextants is the same as for code 2 .  BPE screening of all sextants and pocket depth measurements in sextants scoring 3 at not more than yearly intervals. |
| 4 | A sextant in which the coloured band disappears completely when inserted into the deepest pocket. | Extensive periodontal assessment at outset and post-treatment.  Full probing depth charts required and evaluation of furcation involvements. Use of radiographs for assessment if appropriate.  Treatment as for code 2. Subsequent treatment may include root planing and periodontal surgery |
| * | A sextant in which there is total attachment loss of 7mm or more at any site, or if a furcation can be probed |
