# Supplementary material for: Clinical outcomes of Single-Visit oral Prophylaxis: A practice-based randomised controlled trial
Source: BMC Oral Health. 2011 Dec 28;11:35. doi: 10.1186/1472-6831-11-35 (PMC3280181; doi:10.1186/1472-6831-11-35)
Supplement: Additional File 2 — Sample size calculation information. [file 1472-6831-11-35-S2.DOC]

**Sample Size (Power Calculation)**

There was insufficient literature to adequately inform a precise power calculation for the primary outcome, and similarly the secondary outcomes. A pragmatic approach had to be taken and so a suite of power calculations was carried out by the trial statistician (Table 5):

The main null hypothesis is H_0_: 6=12=24, where 6, 12 and 24 are prevalence of bleeding in each S&P group respectively.

For example, 456 patients are required to consent (assuming 20% loss to follow-up) to achieve a sample size of 121 in each of the 3 groups. This sample size will be sufficient to detect a difference in proportions of bleeding characterized by a Variance of proportions, V=(π_i_-π_0_)^2^ / G of 0.006667 and an average proportion of 50% at the end of the study, with 80% power and a 5% significance level, using χ2 test. This average proportion represents assumption of 40% bleeding rate in 6 months S&P group, 50% bleeding rate in 12 months S&P group and 60% bleeding rate in 24 months S&P group.

In fact, the number of participants recruited was 369 (highlighted in Table 5); sufficient to detect a clinically significant difference in proportions of bleeding assuming 30% bleeding, 45% bleeding, and 60% bleeding in the 6-month, 12-month, and 24-month groups respectively (α=0.01, 90% power)

**Table 5. Sample Size Power calculation**

|  |  | Prevalence of bleeding | | |  |  |  |
| --- | --- | --- | --- | --- | --- | --- | --- |
|  | Power | **6-month Group** | **12-month Group** | **24-month Group** | Number required per group | % lost to follow up | Total number required to consent |
| 0.05 | 80% | 40% | 50% | 60% | 121 | 20% | 456 |
| 0.05 | 90% | 40% | 50% | 60% | 159 | 20% | 597 |
| 0.05 | 80% | 30% | 40% | 50% | 116 | 20% | 435 |
| 0.05 | 80% | 20% | 30% | 40% | 102 | 20% | 369 |
| 0.05 | 80% | 30% | 45% | 60% | 53 | 20% | 201 |
| 0.05 | 90% | 30% | 45% | 60% | 70 | 20% | 264 |
| 0.01 | 80% | 30% | 45% | 60% | 77 | 20% | 291 |
| 0.01 | 90% | 30% | 45% | 60% | 96 | 20% | 360 |

**Reference**

Elashoff JD: nQuery Advisor.Version 5.0 User’s Guide. Los Angeles, CA; 2002.
