# Supplementary material for: Clinical outcomes of Single-Visit oral Prophylaxis: A practice-based randomised controlled trial
Source: BMC Oral Health. 2011 Dec 28;11:35. doi: 10.1186/1472-6831-11-35 (PMC3280181; doi:10.1186/1472-6831-11-35)
Supplement: Additional File 3 — Delivery of standardised oral hygiene advice. [file 1472-6831-11-35-S3.DOC]

**Hygienists and Therapists: Delivery of Oral Hygiene Advice (OHA)**

Hygienists and dental therapists routinely provide scale and polish in family dental practices. They were therefore the preferred provider in this pragmatic trial. All were registered professionals, satisfactorily employed with the dental practices involved in the trial with no concerns regarding their technical abilities. All received instruction regarding the provision of the intervention, and standardised advice. There was no incentive for them to sabotage the trial by providing substandard treatment therefore it was not deemed necessary to check patients post-scaling.

It was inappropriate to withhold oral hygiene advice for the duration of the trial as this would usually be provided. Advice was given to all participants who attended each 6-monthly trial appointments with the hygienist/therapist. This was based upon the Scientific Basis of Oral Health Education[3]. Written information was provided for the hygienists and therapists to read through with the patient and give to them to take away (whilst this is not always part of routine care, leaflets and advice sheets are often distributed by dental care professionals to reinforce homecare.) Advice was not patient-specific and oral hygiene techniques were not demonstrated intra-orally or using models. This enabled a standardised approach to oral hygiene advice.

Written details were as follows:

| ***Careful and effective daily tooth brushing reduces the risk of developing periodontal disease*** |
| --- |
| ***Thoroughly brush all tooth surfaces twice every day, this process should take at least 2 minutes*** |
| ***A gentle scrub technique should be used.*** |
| ***A small, soft to medium texture toothbrush should be used.*** |
| ***Family strength fluoride toothpaste (at least 1,400 ppm of fluoride) should be used.*** |
| ***Avoid excessive pressure as it can increase gum recession and tooth wear.*** |
| ***Dental floss can be used on a daily basis to aid inter-dental cleaning.*** |

**Reference**

Levine R: The scientific basis of oral health education. *Community Dent Health* 2004, 21(2):131-133.
