# Supplementary material for: Clinical outcomes of Single-Visit oral Prophylaxis: A practice-based randomised controlled trial
Source: BMC Oral Health. 2011 Dec 28;11:35. doi: 10.1186/1472-6831-11-35 (PMC3280181; doi:10.1186/1472-6831-11-35)
Supplement: Additional File 4 — Details of training for independent trial examiners in outcome measurements. [file 1472-6831-11-35-S4.DOC]

**Outcome Measurements: Details of training received by independent trial examiners**

Training sessions were held at which 10-15 patients (recruited specifically for this exercise) were examined by the independent examiners under the supervision of a specialist periodontologist.

The specialist described the method by which the measurements should be carried out and advised the sequence in which they should be taken from least invasive – most invasive.

The first trial measurement taken was lingual calculus; a modified Volpe-Manhold Index was used. The tooth with the most lingual calculus evident had a measurement taken in mm along the vertical axis. The independent examiners were advised to round down if the measurement fell between the mm increments marked on the PCP-10 probe.

Measurement of presence of plaque and gingival bleeding were invasive so reproducibility / calibration was not possible. The specialist described the most appropriate method and sequence to undertake the clinical examinations and observed the dentists undertaking trial measurements on the patients. The independent examiners were instructed to use a blunt-ended (PCP-10) probe. This was run gently around the gingival margin of the tooth at angle of approximately 60^o^ in contact with the sulcular epithelium. Minimum axial force was advised to avoid undue penetration into the tissue. The examiners were instructed to probe all index teeth and report visual presence of plaque for recording as they did so. After approximately 30 seconds, the presence of bleeding from the gingival margins of these teeth was recorded.

A BPE (Table 6) was undertaken to identify ineligible patients with BPE greater than 2. BPE was not defined in the protocol as an outcome measure and was used as a screening tool to eliminate ineligible participants at recruitment. Healthy patients with pocketing less than 3.5mm would not normally have full-mouth pocket probing measurements undertaken; hence this was not carried out. More complex indices e.g. gingival or plaque indices [2] are also not routinely used in practice.

Both independent examiners were observed taking all trial measurements and BPE screening and the periodontal specialist was satisfied that they were undertaking the examination in the appropriate manner.

Training sessions were held prior to baseline and follow-up.

**Table 6**. Community Periodontal Index of Treatment Need (CPITN), Basic Periodontal Examination (BPE) and Periodontal Screening and Recording (PSR) codes

| **Code** | **CPITN** | **BPE** | **PSR** |
| --- | --- | --- | --- |
|  | World Health Organization 621 CPITN probe used:  0.5mm diameter ball-ended; section from 3.5mm to 5.5mm coloured black | | |
| **0** | No pocketing or gingival bleeding on probing | No pockets in excess of 3mm deep, no overhanging restorations or calculus, and no bleeding after gentle probing | Coloured area of probe visible. No calculus or defective margins. No bleeding on probing |
| **1** | Gingival bleeding on probing. | No pockets in excess of 3mm deep, no overhanging restorations or calculus, but bleeding occurs after gentle probing. | Coloured area of probe visible.  No calculus or defective margins. Bleeding on probing. |
| **2** | Supra- +/- subgingival calculus or defective restoration margin. | No pockets in excess of 3mm deep, coloured band of probe remains visible, but plaque retentive factors seen or recognised underneath the gingival margin e.g. overhanging restoration margins or subgingival calculus. | Coloured area of probe visible. Calculus or defective margins present. May be bleeding on probing. |
| **3** | Pathological pocket of 4-5mm. | The coloured band of the probe remains partially visible when inserted into the deepest pocket. | Coloured area of probe partially visible. May be calculus or defective restoration margins. May be bleeding on probing. |
| **4** | Pathological pocket of 6mm or greater. | The coloured band disappears completely when inserted into the deepest pocket. | Coloured area of probe not visible. May be calculus or defective restoration margins. May be bleeding on probing. |
| ***** |  | Total attachment loss of 7mm or more at any site, or if a furcation can be probed. | Abnormality such as bone loss in a furcation region, tooth mobility, or gingival recession of 3.5mm in an apical direction from the cement-enamel junction |
| **X** | Only one tooth or no teeth present in the sextant |  |  |

References

Löe H: The Gingival Index, the Plaque Index and the Retention Index System. *Journal of Periodontology* 1967, 38:610 - 616.

Cutress TW, Ainamo J, Sardo-Infirri J: The community periodontal index of treatment needs (CPITN) procedure for population groups and individuals. *Int Dent J* 1987, 37(4):222-233.

British Society of Periodontology: Periodontology in General Practice in the United Kingdom. A Policy Statement.; 2001.

Nasi JH: Background to, and implementation of, the Periodontal Screening and Recording (PSR) procedure in the USA. *International Dental Journal* 1994, 44(5 Supplement 1):585-588.
